# Adaptation of the nutrition care process for metabolic diseases in the Mexican population

**DOI:** 10.3389/fnut.2025.1513747

**Published:** 2025-01-28

**Authors:** Magdalena Sevilla-González, Ailema González-Ortiz, María Victoria Landa-Anell, Marco A. Melgarejo-Hernández, Ana Teresa Arias-Marroquín, Fabiola Mabel Del Razo-Olvera, Berenice Monserrat Román-Calleja, Ana Victoria Monreal-Lugo, Angélica J. Martin-Vences, Karime Haua-Navarro, Angeles Espinosa-Cuevas

**Affiliations:** ^1^Clinical and Translational Epidemiology Unit, Mongan Institute, Massachusetts General Hospital, Boston, MA, United States; ^2^Department of Medicine, Harvard Medical School, Boston, MA, United States; ^3^Programs in Metabolism and Medical & Population Genetics, The Broad Institute of MIT and Harvard, Cambridge, MA, United States; ^4^Department of Medical Epidemiology and Biostatistics, Karolinska Institutet, Stockholm, Sweden; ^5^Translational Research Center, Instituto Nacional de Pediatría, Mexico City, Mexico; ^6^Centro de Atención Integral del Paciente con Diabetes (CAIPaDi), Instituto Nacional de Ciencias Médicas y Nutrición Salvador Zubirán, Mexico City, Mexico; ^7^Department of Endocrinology and Metabolism, Lipid Clinic, Instituto Nacional de Ciencias Médicas y Nutrición Salvador Zubirán, Mexico City, Mexico; ^8^Department of Epidemiological Surveillance, Dirección de Nutrición, Instituto Nacional de Ciencias Médicas y Nutrición Salvador Zubirán, Mexico City, Mexico; ^9^Metabolic Diseases Research Unit, Instituto Nacional de Ciencias Médicas y Nutrición Salvador Zubirán, Mexico City, Mexico; ^10^Department of Gastroenterology, Instituto Nacional de Ciencias Médicas y Nutrición Salvador Zubirán, Mexico City, Mexico; ^11^Facultad Mexicana de Medicina, Universidad La Salle, Mexico City, Mexico; ^12^Colegio Mexicano de Nutriólogos, Mexico City, Mexico; ^13^Nephrology and Mineral Metabolism Department, Instituto Nacional de Ciencias Médicas y Nutrición Salvador Zubirán, Mexico City, Mexico

**Keywords:** nutrition therapy, educational, diabetes mellitus, obesity, metabolic diseases, population health

## Abstract

**Background:**

The Nutrition Care Process (NCP) is a systematic framework designed to enhance the quality of nutrition care. Given the high prevalence of metabolic diseases in Mexican population, there is a critical need for tailored nutrition care strategies.

**Objective:**

We aim to describe the adaptation of the NCP to manage metabolic diseases in Mexican individuals.

**Methods:**

Our adaptation included a comprehensive literature review of clinical nutrition guidelines, by a structured consultation with experts to ensure clinical setting-specific and culturally appropriate modifications. A team of registered dietitians from two tier 3 hospitals, each with over five years of experience in metabolic disease management, customized the NCP’s four core steps—assessment, diagnosis, intervention, and monitoring—to meet the specific needs of the Mexican population.

**Results:**

We adapted the NCP to manage five common metabolic disorders: obesity, type 2 diabetes, kidney disease, metabolic dysfunction-associated steatotic liver disease, and dyslipidemia. Each step of the NCP was complemented by the development of educational materials designed to (1) enhance awareness of disease risk, (2) broaden their knowledge of nutritional management, and (3) provide tailored strategies for developing personalized action plans. The adapted NCP was implemented in clinical and research settings and the materials were documented as an online publication to facilitate widespread dissemination.

**Conclusion:**

Our adaptation represents a significant advancement in the use of structured tools for nutrition care in Mexican populations, who face disproportionately high rates of metabolic diseases. Further research is needed to assess the effectiveness of this approach in clinical settings.

## Introduction

Metabolic diseases, including obesity, Type 2 Diabetes Mellitus (T2DM), chronic kidney disease (CKD), Metabolic Dysfunction-Associated Steatotic Liver Disease (MASLD), and dyslipidemia, represent a significant global health challenge, particularly for Hispanic populations, where the rates of these disorders are disproportionately high. In Mexico, the 2022 National Health and Nutrition Survey (ENSANUT) indicates that 75.2% of Mexican adults are classified as overweight or obese (1). In 2021, this alarming rate was linked to 118,000 deaths attributable to elevated body mass index (BMI), with elevated BMI accounting for 55% of T2DM-related deaths and 41.1% of deaths from CKD (2). Dyslipidemia also significantly affects the adult population, with approximately 50% presenting some form of lipid disorder (3). Moreover, MASLD (previously known as NAFLD and later as MAFLD), represents a significant and growing public health challenge in Mexico with a prevalence of 17–41.3%, this condition is intricately linked to the country’s rising rates of obesity, T2DM, and metabolic syndrome (4). Despite its impact, accurate diagnosis of MASLD remains limited due to diagnostic challenges (5). In 2021, the prevalence of CKD was reported at 9,184.9 cases per 100,000 inhabitants, largely driven by T2DM, the leading cause of CKD. T2DM contributed to 69,052 deaths (95% CI = 60,412–77,991) across all age groups in Mexico (6). Furthermore, the prevalence of T2DM was estimated at 18.3% among adults aged 20 years and older, equating to around 14.6 million individuals (7).

Unhealthy dietary habits, particularly the consumption of sugary drinks, processed meats, saturated fats, and general caloric imbalance, are the most significant modifiable factors associated with metabolic diseases, contributing to 27.3% of Disability-Adjusted Life Years (DALY) lost (8). Effective management of these diseases requires addressing dietary risk factors. Nutritional Medical Treatment (NMT) is a crucial component emphasized in various consensus statements for its integration into multidisciplinary teams managing metabolic conditions. Guidelines recommend implementing NMT through comprehensive nutritional education programs aimed at promoting self-care. This approach should be consistently reinforced across different life contexts to achieve metabolic control, reduce complications, and improve quality of life (3, 9). International guidelines advocate for nutrition professionals to utilize evidence-based NMT to address nutritional issues at both individual and population levels.

The Nutrition Care Process (NCP), introduced by the Academy of Nutrition and Dietetics in 2003, provides a valuable framework for implementing NMT across various clinical settings (10–13). Despite its potential, the NCP is underutilized in nutrition service centers, primarily due to barriers such as inefficient staffing, limited time for documentation, and the lack of necessary infrastructure, including supportive electronic health record systems (14–17). While the NCP has been shown to enhance nutritional care quality in chronic metabolic diseases (18–20). most studies focus on non-Latin American settings. This gap indicates different health systems, food cultures, accessibilities, and metabolic disease rates, such as those in Mexico. Research supports the effectiveness of culturally adapted programs to improve lifestyle intervention adherence among Latinos in the US (21), underscoring the need for tools that simplify the NCP’s implementation in environments reflecting the unique characteristics of the Mexican population, especially concerning prevalent metabolic disorders, particularities of the health system and food culture. In this study, we aimed to describe the adaptation of the NCP for managing five metabolic diseases— Individuals with Obesity at Risk of Type 2 Diabetes (ORT2D), T2DM, CKD, MASLD, and dyslipidemia—in Mexican individuals. The objectives of the adaptation involved prioritizing specific evaluation items and nutritional diagnoses for each disease, creating educational materials, summarizing clinical guidelines for nutritional management, and recommending tailored monitoring tables for each pathology.

## Methods

### Adaptation team

The selection criteria for the adaptation team for each disease included extensive experience in the nutrition care of metabolic diseases and significant research involvement. Seven dietitians from one tier 3 hospital and another from a different tier 3 hospital were selected based on their diverse clinical expertise and perspectives. All chosen dietitians were required to have at least a master’s degree and active participation in research protocols in their field, ensuring a rigorous evaluation of the quality of the evidence presented. Additionally, each dietitian had a minimum of five years of experience and was actively involved in their respective specialty departments. All members had also completed at least one training course on NCP methodology. The teams were organized by disease focus—ORT2D, T2DM, CKD, MASLD, and dyslipidemia—to optimize the adaptation process. The tier 3 hospitals function as referral centers, directing patients from various regions to specialized medical and nutritional care and also offering areas for non-specialized, general treatment. This structure accommodates a broad spectrum of the country’s healthcare needs and ensures a wide representation of the population.

**Figure 1:**
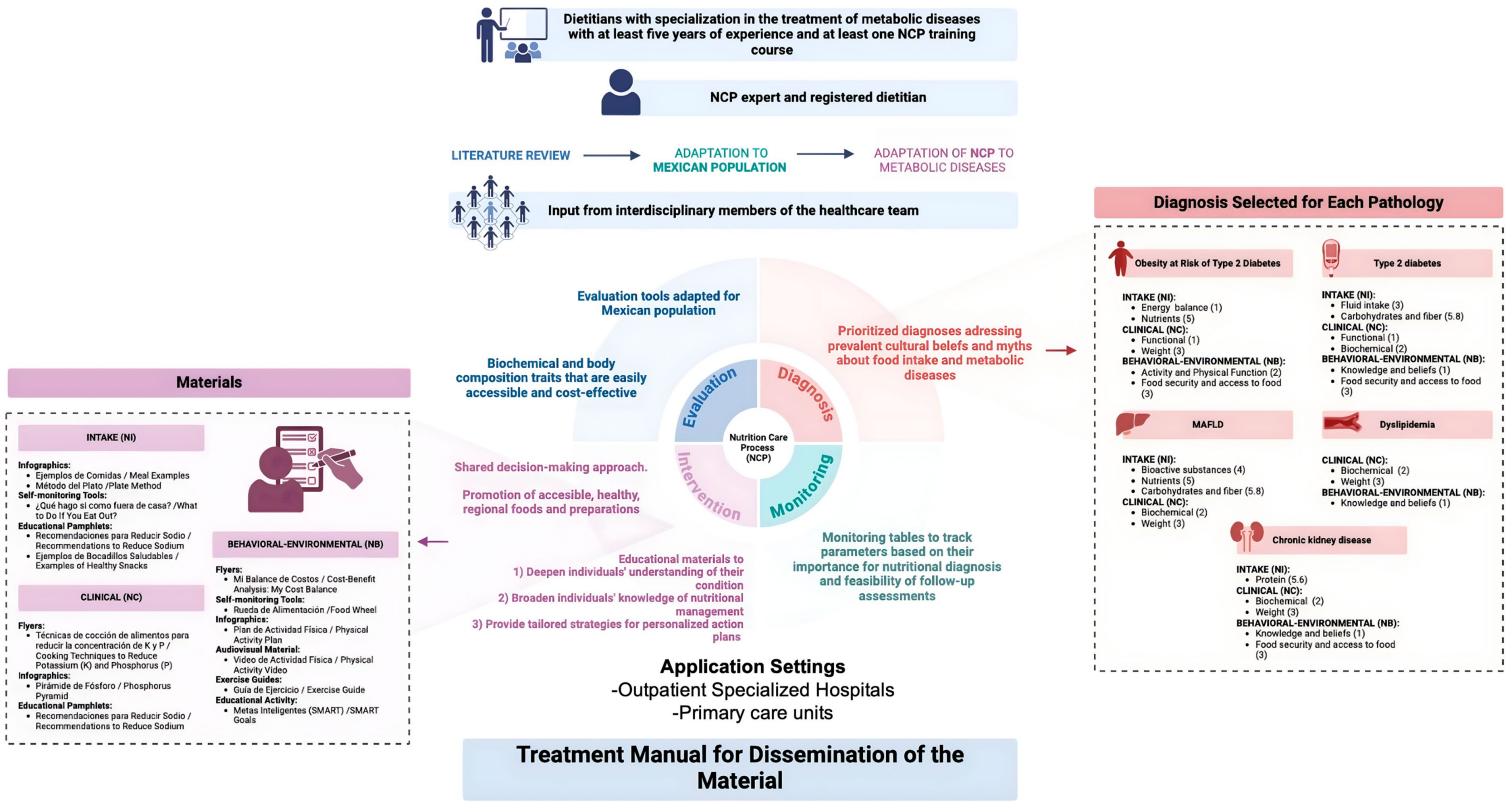
Summary of the adaptation of the nutrition care process in metabolic diseases in Mexican population. Created with BioRender.com.

The adaptation was done in three steps: 1.- Literature review, 2.- Adaptation to Mexican population, 3.- Adaptation of NCP to metabolic diseases and development of educational materials (Figure 1).

### Critical literature review

Each team gathered scientific literature pertinent to their specific pathology. We conducted a literature review to gather the most current information on the epidemiological status and clinical guidelines for the nutritional management of five pathologies. Clinical guidelines were obtained from national and international medical societies, and we accessed relevant information through PubMed database. There was no restriction on the publication year for the literature search, although the most current clinical guidelines and research studies published more than five years ago were included, provided they were highly relevant to the topic. To standardize the literature search, each researcher used keywords and MeSH terms, excluding only reports and case series. We also prioritized studies focusing on the Mexican population.

### Adapting nutrition guidelines for the Mexican population

Each team facilitated discussions with the aim to compare the recommendations outlined in the guidelines with their practical implementation in real clinical settings, identifying any discrepancies or gaps. This approach was designed to reveal potential deficiencies in the application of guidelines within real-world scenarios.

### Adapting the NCP for metabolic diseases

We carefully reviewed the evaluation items, aligning them with relevant literature to include only those pertinent or most used in the treatment of metabolic diseases. Similarly, we prioritized common diagnoses observed in clinical practice for adaptation. Regarding interventions, each team documented guidelines-based suggestions tailored to our population. To facilitate the educational intervention, we assembled a toolbox of educational resources. This helped to simplifying the explanation of nutritional and disease concepts. Some of the adapted materials were piloted implemented in an interdisciplinary program for treating individuals with T2DM, as well as in nutrition clinics for patients with dyslipidemia, MASLD, and renal diseases. This implementation facilitated valuable feedback from both patients and dietitians. Lastly, we developed pathology-specific monitoring tables, incorporating suggested monitoring times from literature and clinical practice.

The implementation of the educational materials was fully approved by the Ethics and Research Committee of the Instituto Nacional de Ciencias Médicas y Nutrición Salvador Zubirán and was registered at ClinicalTrials.gov (NCT02836808). Written informed consent was obtained from each participant. Research was conducted according to the tenets of the Helsinki Declaration of Human Studies principles.

### Integrating interdisciplinary for adaptation

Following the framework of the NCP methodology, each adaptation team sought input from interdisciplinary team members, all of whom were part of the same clinical team within the institution. These team members offered valuable insights into the patient treatment context and provided tools to identify referral signals. This collaborative approach ensures a comprehensive overview of patient needs for an effective care coordination.

### Expert consultation for NCP adaptation

Throughout the adaptation process, our team received invaluable support from an NCP expert—a registered dietitian with over a decade of experience in NCP methodology. With a robust background, including completion of courses at the Academy of Nutrition and Dietetics and years of experience conducting training sessions. Regular meetings were held with the expert to address queries and ensure clarity. The final report was reviewed and edited by the NCP expert, ensuring alignment with NCP standards. We documented our results and process in a document, which was published as a book.

## Results

Our literature review encompassed 370 articles, including 25 clinical practice guidelines. Two of these guidelines specifically focused on exercise and physical activity [Guides 1 (22) and 24 (23), as detailed in Table 1] for managing obesity and dyslipidemia. The majority of the guidelines supported the implementation of the NCP across various settings, primarily in primary care for metabolically stable patients. They provided a framework for outpatient management, with limited recommendations for inpatient care [Guides 8 (24) and 15 (25) in Table 1].

**Table 1:** Clinical guidelines used in the development of the adaptation of the NCP model in Mexican population.

Pathology	Guidelines
Obesity at risk of type 2 diabetes	Exercise Testing and Prescription; 2021 (22). Obesity Treatment in Primary Care; 2016 (51).
Type 2 diabetes	AHA/ACC/TOS Management of Overweight and Obesity in Adults; 2014 (52). Type 1 and Type 2 Diabetes in Adults; 2017 (53).
MASLD	JSGE. Nonalcoholic fatty liver disease/nonalcoholic steatohepatitis; 2015 (54). Nonalcoholic fatty liver disease/nonalcoholic steatohepatitis; 2015 (54). NAFLD; 2018 (55). EASL, EASD, EASO. Management of non-alcoholic fatty liver disease; 2016 (56). ESPEN Liver disease; 2019 (24).
Chronic kidney disease	K/DOQI Nutrition in chronic renal failure; 2001 (57). EBPG Nutrition; 2007 (58). KDIGO 2012 Evaluation and Management of Chronic Kidney Disease (59). KDIGO 2020 Diabetes Management in Chronic Kidney Disease (60). KDIGO 2021 Management of Blood Pressure in Chronic Kidney Disease (61). KDOQI Nutrition in CKD: 2020 (62). ESPEN Enteral nutrition: Adult renal failure; 2006 (25). K/DOQI Chronic kidney disease: evaluation, classification, and stratification. 2002 (63, 64). Dietary Guidelines for Americans; 2010 (64).
Dyslipidemia	AHA/ ACC Lifestyle management to reduce cardiovascular risk; 2013 (65). AHA/ACC/TOS The Obesity Society; 2014 (52). Management of Dyslipidemia and Prevention of Cardiovascular Disease; 2017 (66).
	ESC/EAS Management of dyslipidemias. 2011 (67). Scientific report of the 2015 Dietary Guidelines Advisory Committee (68). The 2015 US Dietary Guidelines (69). Physical activity for adults; 2010 (23).

AHA; American Heart Association, ACC; American College of Cardiology, TOS; the Obesity Society, JSGE; Japanese Society of Gastroenterology, NAFLD; Non-alcoholic fatty liver disease, EASL; European Association for the Study of the Liver, EASD; European Association for the Study of Diabetes, EASO; European Association for the Study of Obesity, AASLD; American Association for the Study of Liver Diseases, ESPEN; European Society for Clinical Nutrition and Metabolism, K/DOQI; Kidney Disease Outcomes Quality Initiative, KDIGO; Kidney Disease Improving Global Outcomes, ESC; European Society of Cardiology, EAS and European Atherosclerosis Society.

### Evaluation

In selecting evaluation items, we focused on the most commonly used and critical metrics for each pathology, prioritizing biochemical and body composition traits that are accessible and cost-effective. We developed medical histories in two formats—simplified and detailed—to accommodate diverse clinical settings and address the time constraints faced by dietitians in both primary care and tier 3 hospitals within the Mexican health system. Our aim was to streamline the evaluation process, enhancing efficiency and practicality for healthcare professionals. Furthermore, we incorporated Mexican-adapted evaluation tools, such as the Malnutrition Inflammation Score for CKD (26), vector analysis and body composition assessments in hemodialysis patients (27) and two formulas for estimating energy expenditure specific to the Mexican population (28, 29).

### Diagnostics

For diagnostics, we identified the conditions most commonly observed in individuals with the specified metabolic pathologies. Special attention was given to addressing prevalent cultural beliefs and myths related to food intake that negatively impact the understanding and management of these diseases. Recognizing that individuals in this population can be at risk for metabolic diseases even at lower BMI values (30, 31), we emphasized the importance of considering alternative parameters beyond BMI for diagnosis. Where feasible (in-hospital or clinical research settings), more precise anthropometric and clinical measures like body fat percentage were utilized to provide a better assessment of health status. The most common diagnostics for each pathology are detailed in Table 2.

**Table 2:** Common nutritional care process diagnosis selected for each pathology.

Metabolic disease	Diagnosis	Code	Number
Intake (NI)			
ORT2D, MASLD	Energy balance (1)		
	Excessive energy intake	NI-1.3	10,635
ORT2D, T2DM	Fluid intake (3)		
	Excessive fluid intake	NI-3.2	10,650
ORT2D, T2DM, MASLD	Bioactive substances (4)		
	Excessive intake of food additives	NI-4.2.6	11,083
ORT2D, T2DM, MASLD	Nutrients (5)		
	Inadequate (suboptimal) energy-protein intake	NI-5.2	10,658
	Nutrient imbalance	NI-5.4	10,660
CKD, T2DM	Protein (5.6)		
	Inadequate protein intake	NI-5.6.1	10,666
	Excessive protein intake	NI-5.6.2	10,667
ORT2D, T2DM, MASLD	Carbohydrates and fiber (5.8)		
	Excessive carbohydrate intake	NI-5.8.2	10,671
	Deficient fiber intake	NI-5.8.5	10,675
Clinical (NC)			
ORT2D, T2DM	Functional (1)		
	Altered gastrointestinal function	NC-1.4	10,757
ORT2D, T2DM, MASLD, CKD	Biochemical (2)		
	Altered nutrition-related laboratory values (specify)	NC-2.2	10,760
ORT2D, T2DM, CKD, Dyslipidemia	Weight (3)		
	Overweight/obesity	NC-3.3	10,766
	Overweight, adult or pediatric	NC-3.3.1	10,767
	Type I obesity	NC-3.3.3	10,769
	Type II obesity	NC-3.3.4	10,818
	Type III obesity	NC-3.3.5	10,819
Behavioral-environmental (NB)			
ORT2D, T2DM, CKD, MASLD	Knowledge and beliefs (1)		
	Insufficient knowledge in food and nutrition topics	NB-1.1	10,773
	Not prepared for lifestyle/diet change	NB-1.3	10,775
	Deficit in self-monitoring	NB-1.4	10,776
	Insufficient adherence to nutritional recommendations	NB-1.6	10,778
	Undesirable food choices	NB-1.7	10,779
ORT2D, T2DM, CKD	Activity and Physical Function (2)		
	Physical inactivity	NB-2.1	10,782
	Poor nutritional quality of life	NB-2.5	10,786
ORT2D, T2DM, CKD	Food security and access to food (3)		
	Limited access to food	NB-3.2	10,790
	Others (NO)		
	No nutritional diagnosis at this time	NO-1.1	10,795

NI: Intake domain; NC: Clinical domain; NB: Behavioral-Environmental domain; ORT2D: Individuals with Obesity at Risk of Type 2 Diabetes; T2DM: Type 2 Diabetes Mellitus; CKD: kidney disease; MASLD: Metabolic Dysfunction-associated Steatotic Liver Disease.

### Intervention

#### Dietetic

We tailored dietary modifications to consider the availability of regional foods and the specific nutritional needs of the Mexican population (32). Additionally, these modifications were integrated into dietary recommendations for outpatient nutritional counseling, ensuring they are practical and applicable in real-world settings. Key macronutrient adjustments were made in sources of carbohydrates, proteins, and fatty acids. In Mexico, corn is a primary carbohydrate source, while chicken, eggs and beans are significant protein sources (33–35). We developed strategies to highlight the availability and accessibility of healthy food options, challenging the prevailing belief that healthy eating is expensive and hard to reach (36). Our objective was to promote healthier versions of regional foods through nutritious preparations, positioning these healthier choices as the most convenient option.

#### Counseling

We adopted a shared decision-making framework, wherein health-related decisions are collaboratively made between the patient and a health professional (37, 38). Specifically, treatment options were discussed between dietitians and patients, with a strong emphasis on adapting these options to be more accessible for the patients. This approach enabled patients to choose the treatment strategies they found most suitable. Additionally, when setting goals, we utilized the SMART method (39) to ensure they were specific, measurable, achievable, relevant, and time-bound, thereby enhancing the effectiveness and achievability of the goals set during counseling.

#### Educational materials

Our educational resources were designed to address three critical areas: healthy eating, physical activity, and disease risk awareness. The materials aimed to enhance individual’s awareness of disease risks, enhance their knowledge of nutritional management, and provide tailored strategies for personalized action plans. We ensured that all materials were written in clear, accessible language and featured engaging designs. They were produced in various formats, including infographics, educational pamphlets, recipes, exercise guides, and self-monitoring tools. Infographics and pamphlets provided concise, clear information on nutrition and health, offering guidelines for balanced diets and disease-specific advice. Interactive educational activities were incorporated to boost participant engagement and understanding of their conditions. Recipe books highlighted practical, balanced meal examples, focusing on regional recipes and locally accessible ingredients. Audiovisual materials were utilized in training sessions and workshops to reinforce learning. A summary of each educational material, its focus area, and its alignment with diagnoses from the NCP is detailed in Table 3.

**Table 3:** Materials, learning goal, NCP diagnoses and type of material.

Material name (Spanish/English)	Learning goal	NCP Diagnoses	Type of material
Healthy eating			
De la Idea a la Acción/From Idea to Action (Personalized Meal Planning)	Step-by-step guide to creating a personalized meal plan, including food group equivalents and ingredients.	Intake (NI): Excessive energy intake (NI-1.3), Nutrient imbalance (NI-5.4)	Infographics
¿Cuánta agua llevas?/How Much Water Have You Drunk?	Visual guide to help increases water intake.	Intake (NI): Inadequate fluid intake NI-3.1	Infographics
Plato Saludable y Uso de las Manos/Healthy Eating Plate and Using Hands to Measure	Visual guide for balanced meals using hands to measure portions.	Intake (NI): Excessive energy intake (NI-1.3), Nutrient imbalance (NI-5.4)	Infographics
Método del Plato/Plate Method	Visual guide for balanced meals focusing on appropriate portions of proteins, carbohydrates, and fats.	Intake (NI): Excessive energy intake (NI-1.3), Nutrient imbalance (NI-5.4)	Infographics
Ejemplos de Comidas/Meal Examples	Example menus for balanced daily meals, focusing on portion control and nutrient variety.	Intake (NI): Inconsistent carbohydrate intake (NI-5.8.4), Nutrient imbalance (NI-5.4)	Infographics
Consumo de Grasas Saludables/Consumption of Healthy Fats	Information on selecting healthy fats, such as monounsaturated and polyunsaturated fats, and avoiding trans and saturated fats.	Intake (NI): Deficient lipid intake (NI-5.5.1), Excessive lipid intake (NI-5.5.2)	Infographics
Semáforo de Productos/Product Traffic Light	Tool to classify foods based on their energy density, encouraging low-density options for better health.	Behavioral-Environmental (NB): Undesirable food choices (NB-1.7)	Flyers
Técnicas de cocción de alimentos para reducir la concentración de K y P/Cooking Techniques to Reduce Potassium (K) and Phosphorus (P)	Cooking Techniques to Reduce phosphorus and potassium.	Clinical (NC): Chronic kidney disease (NC-1.4)	Flyers
Rueda de Alimentación/Food Wheel	Tool to assess performance in different aspects of life that impact nutrition, such as emotional eating, exercise, and body image.	Behavioral-Environmental (NB): Disordered eating pattern (NB-1.5), Physical inactivity (NB-2.1)	Self-monitoring Tools
¿Qué hago si como fuera de casa?/What sould I do If I Eat Out?	Tips for choosing healthier options when dining out, including what to prefer and what to avoid.	Intake (NI): Excessive energy intake (NI-1.3). Behavioral-Environmental (NB): Undesirable food choices (NB-1.7)	Self-monitoring Tools
Talleres Interactivos/Interactive Workshops	Engaging sessions to educate about healthy eating habits, physical activity, and overall lifestyle changes.	Depending on specific NCP diagnoses	Educational Activity
Recomendaciones para Reducir Sodio/Recommendations to Reduce Sodium	Guidelines on reducing sodium intake by choosing healthier alternatives and avoiding high-sodium products.	Intake (NI): Excessive sodium intake (NI-5.10.7). Clinical (NC): Hypertension	Educational Pamphlets
Ejemplos de Bocadillos Saludables/Examples of Healthy Snacks	Examples of balanced snacks that combine carbohydrates with proteins or fats for sustained energy and satiety.	Intake (NI): Inadequate (suboptimal) energy-protein intake (NI-5.2)	Educational Pamphlets
Ejemplos de Comidas/Meal Examples	Example menus for balanced daily meals, focusing on portion control and nutrient variety.	Intake (NI): Inconsistent carbohydrate intake (NI-5.8.4), Nutrient imbalance (NI-5.4)	Educational Pamphlets
Physical activity			
Plan de Actividad Física/Physical Activity Plan	Guidelines to incorporate physical activity into daily routines with tips on frequency, intensity, and types of exercises.	Behavioral-Environmental (NB): Physical inactivity (NB-2.1), Excessive physical activity (NB-2.2)	Infographics
Video de Actividad Física/Physical Activity Video	Demonstration of exercises and guidelines for incorporating physical activity into daily routines.	Behavioral-Environmental (NB): Physical inactivity (NB-2.1), Excessive physical activity (NB-2.2)	Audiovisual Material
Guía de Ejercicio/Exercise Guide	Detailed guide on different exercises, their benefits, and how to perform them safely.	Behavioral-Environmental (NB): Physical inactivity (NB-2.1), Excessive physical activity (NB-2.2)	Exercise Guides
(3) Disease risk awareness			
Pirámide de Fósforo/Phosphorus Pyramid	Visual guide to managing phosphorus intake for kidney health.	Clinical (NC): Chronic kidney disease (NC-1.4)	Infographics
Mi Balance de Costos/Cost–Benefit Analysis: My Cost Balance	Tool to weigh the advantages and disadvantages of making lifestyle changes.	Behavioral-Environmental (NB): Not ready to make diet or lifestyle changes (NB-1.3)	Flyers
Comer por Ansiedad/Eating Due to Anxiety	Strategies to address emotional eating, including identifying triggers and replacing harmful habits with healthier actions.	Behavioral-Environmental (NB): Disordered eating pattern (NB-1.5), Emotional eating (NB-1.7)	Flyers
¿Cómo como?/Food Diary: How Do I Eat?	Food diary to record meals, hunger levels, and emotions associated with eating.	Intake (NI): Inconsistent carbohydrate intake (NI-5.8.4), Deficit in self-monitoring (NB-1.4)	Self-monitoring Tools
Metas Inteligentes (SMART)/SMART Goals	Framework to set specific, measurable, achievable, realistic, and time-bound goals.	Behavioral-Environmental (NB): Insufficient adherence to nutritional recommendations (NB-1.6)	Educational Activity
¿Sabes que te estas comiendo?/Do You Know What You Are Eating? Reading Labels	Information on how to read food labels to make healthier choices.	Behavioral-Environmental (NB): Undesirable food choices (NB-1.7)	Educational Pamphlets

NI: Intake domain; NC: Clinical domain; NB: Behavioral-Environmental domain; Infographics: Visual representations summarizing key information on nutrition and health topics. Flyers: Short, concise materials focusing on key health messages. Self-monitoring Tools: Tools for tracking dietary intake, physical activity, and other health metrics. Educational activity: Visual materials to reinforce key messages about healthy eating and lifestyles. Educational Pamphlets: Booklets and brief documents on specific healthy eating topics. Audiovisual Material: Videos and multimedia presentations used in workshops and training sessions. Exercise Guides: Instructions and tips for physical activity and exercise.

##### Interdisciplinary

For the pathologies ORT2D and MASLD, we engaged with physicians specializing in these disorders to gain deeper insights into the integration of nutritional and medical treatments. Specifically, for ORT2D, feedback from a psychology expert was incorporated, detailing tools designed to enhance patient adherence to nutritional treatments. Recognizing the high demand for treatment among these patient populations, we also explored the implementation of electronic tools aimed at improving adherence to dietary recommendations, which could facilitate more efficient patient management and outcome tracking (40).

### Monitoring

We developed monitoring tables to systematically track the most relevant clinical and behavioral parameters across subsequent visits. The selection of these parameters was based on their critical role in nutritional diagnosis and their feasibility for follow-up assessments in real world-settings. Given the logistical and economic challenges faced by this population, including limited appointment availability and transportation difficulties, we prioritized parameters that are practical and cost-effective to monitor. Additionally, considering the high costs of clinical tests for patients and institutions, optimizing these parameters is essential for resource management and effective monitoring. To address and mitigate barriers during treatment, we employed strategies derived from documented experiences of individuals with T2DM (41) in similar settings, facilitating improved adherence and outcomes.

### Implementation of adapted NCP in diverse settings

The adapted NCP was implemented across various clinical and research settings, each tailored to meet specific needs and objectives. In research settings, the adaptation was primarily applied within lifestyle intervention protocols for individuals at risk of type 2 diabetes. These protocols utilized the adapted NCP to guide personalized nutritional interventions, emphasizing evaluation, nutrition diagnosis, and rigorous documentation and follow-up, as detailed in our recent studies (40, 42).

In contrast, the clinical implementation focused on comprehensive care programs at the National Institute of Medical Sciences and Nutrition Salvador Zubirán in Mexico City. Here, the adapted NCP was integrated into routine patient care for managing Type 2 Diabetes Mellitus (T2DM), supporting patients in achieving and maintaining health goals through structured nutritional interventions within a multidisciplinary team framework (11, 41, 43–45).

Additionally, departments specializing in dyslipidemia, gastroenterology, and renal diseases at the same institute adopted the adapted NCP materials, including educational resources and nutritional diagnostic processes, into their clinical practices. Evidence indicates feasibility of implementation and differences in the effectiveness of the adapted NCP across clinical and research settings. In research protocols, the adapted approach not only standardized lifestyle interventions among dietitians but also enhanced the monitoring of nutritional care. In clinical settings, the adaptation has contributed to identify the barriers to adherence to a nutritional plan and strategies to overcome them in patients with T2D (41). It also supported the stratification of individuals based on their nutritional diagnosis, enabling more tailored and effective nutritional treatment within an interdisciplinary program (11). These findings suggest that the implementation of culturally and disease-adapted nutritional interventions can significantly improve nutrition practice across diverse settings.

### Development of a resource for disseminating the adapted material

To disseminate our findings, we compiled them into a comprehensive book. The book is structured into six chapters: the first chapter outlines the general principles of the NCP, while the subsequent chapters focus on its implementation for specific pathologies. Where applicable, subchapters on interdisciplinary treatment approaches are also included. Each chapter begins with an introduction to the disease, followed by a presentation of its global and regional epidemiology. The chapters detail how the steps of the NCP—evaluation, diagnosis, intervention, and monitoring—are tailored for each pathology. To enhance clarity and usability, abbreviated tables summarizing the most frequently used NCP terms for each pathology are included. A detailed checklist of the sections for each pathology can be found in Supplementary Table S1. Furthermore, we developed a comprehensive toolbox of educational resources to support the effective implementation of the NCP. Each chapter also features a clinical case example, demonstrating the practical application of the materials in real-world scenarios. The book is available as an online publication at the following link: https://www.amazon.com.mx/proceso-atencion-nutricia-enfermedades-metab%C3%B3licas-ebook/dp/B0BPJKNDHZ. The content of the book was reviewed and subsequently approved by the publications committee of the Mexican Society of Endocrinology and Nutrition, ensuring the integrity and accuracy of the information presented.

## Discussion

We adapted the NCP to address five prevalent metabolic diseases in the Mexican population. The material provides a standardized framework for the various NCP steps—diagnosis, evaluation, nutritional intervention, and monitoring—to ensure consistent care across different settings and providers. Each step of the NCP was meticulously customized to these conditions, including the development of targeted educational materials designed to (1) increasing individuals’ disease risk awareness, (2) broaden their knowledge of nutritional management, and (3) provide tailored strategies for developing personalized action plans. This tailored approach was successfully implemented in both research and clinical environments. The educational materials created are documented in an online book publication, serving as a valuable resource for health professionals.

The NCP has been adapted and successfully implemented in diverse settings and geographical locations, though several barriers to its adoption have been noted. In the Philippines, authors at a level 3 hospital emphasized the need for enhanced support from institutions, professional organizations, and policymakers to facilitate implementation (46). In Saudi Arabia, although most dietitians are familiar with the NCP and confident in its application, it has not yet been adopted as a standard practice in hospitals (47). Australian dietitians, prior to implementation, reported barriers including a lack of knowledge, support, training, and resources, while those already using the NCP identified heavy workloads and work status as obstacles (48). Lövestam et al. stressed the necessity for context-specific strategies to address local challenges and improve dietetic support (16).

The primary barriers to NCP implementation have been identified as inefficient staffing, limited time for completing necessary documentation, and the lack of supportive infrastructure, such as electronic health record systems (16, 17). In Switzerland, the integration of NCP documentation into electronic patient records has helped strengthen the linkage between assessment and nutrition diagnosis, although the frequent selection of similar nutrition problems by dietitians’ points to an ongoing challenge in adopting critical thinking (49). Additionally, Chen et al. explored mobile applications as a means to enhance the efficiency of the nutrition care process, potentially freeing up more time for dietetic counseling (50), found that apps could improve the efficiency of the nutrition care process, allowing more time for dietetic counseling. While these apps show promise in complementing dietetic care, they cannot replace direct practice. In Mexico, where the demand for healthcare services related to metabolic diseases is rapidly increasing, the adoption of electronic systems and tools that streamline healthcare management and reduce commute times to hospitals presents an attractive opportunity for further exploration.

Our study represents a pioneering effort in adapting the NCP for metabolic diseases within a Latin-American context, marking the first such implementation across diverse clinical and research settings in the region. This adaptation not only addresses a significant gap in the literature but also provides a practical framework for healthcare professionals in similar settings. However, several limitations warrant mention. First, the literature review that underpinned the adaptation process was conducted several years ago. While it incorporated the most relevant and up-to-date references available at that time, some of the references may now appear outdated, potentially overlooking recent advances in the management of metabolic diseases. Despite this, we have made efforts to update critical aspects, such as the nomenclature and clinical nutrition management of the diseases, to reflect current standards and practices. Second, the scope of the literature review was primarily confined to PubMed. This decision was made due to PubMed’s comprehensive coverage of biomedical literature, which aligned well with our study’s focus on metabolic diseases. However, this approach, while thorough within its specified domain, inevitably limited the breadth of our review by excluding potentially valuable insights from broader multidisciplinary sources available in other databases such as Web of Science or ScienceDirect. This limitation may have restricted our perspective and lessened the comprehensiveness of the adaptation process.

The effective implementation of the NCP in Mexican healthcare settings demands a well-established set of skills, robust institutional support, and specific practices to ensure its adoption, sustainability, and impact. Dietitians treating metabolic diseases often encounter significant challenges, including high workloads and budget constraints, which can impede the adoption of new strategies requiring additional training or resources. Moreover, the role of dietitians is not consistently defined within institutional decision-making processes, complicating the implementation of standardized care protocols. Successful implementation relies on allocating dedicated time for practitioners to familiarize themselves with the NCP, providing ongoing training to maintain and improve skills, and securing strong support and leadership from management and department leaders. These elements represent both challenges and opportunities for institutions to advocate for programs that demonstrate the benefits of such changes, thereby supporting the sustained impact of the NCP. The adapted NCP discussed in this study marks substantial progress in creating targeted tools to enhance nutritional counseling practices in populations severely affected by metabolic diseases. A standardized NCP framework not only promotes consistent practice but also provides a solid basis for evaluating the effects of nutrition care on patient health outcomes. Achieving this, however, requires active participation and commitment from both institutions and healthcare professionals. Future research should focus on validating the effectiveness of these tools in real-world settings exploring their implementation alongside electronic systems to optimize service delivery.

## Conclusion

We adapted the NCP for five metabolic diseases, incorporating assessment tools, dietary recommendations, and educational materials specifically tailored to the Mexican population. This comprehensive framework is designed to enhance professional nutrition practice. The adaptation is particularly significant for the Mexican population, which faces disproportionately high rates of metabolic diseases and associated treatment challenges. Who face disproportionately high rates of metabolic diseases and related treatment challenges. Future research is needed to evaluate the effectiveness of this adapted NCP in clinical settings and to explore its integration with electronic health records and mobile apps to facilitate implementation within health systems.

## Data Availability

The raw data supporting the conclusions of this article will be made available by the authors, without undue reservation.
