# Supplementary material for: Adaptation of the nutrition care process for metabolic diseases in the Mexican population
Source: Front Nutr. 2025 Jan 28;12:1513747. doi: 10.3389/fnut.2025.1513747 (PMC11841437; doi:10.3389/fnut.2025.1513747)
Supplement: Supplementary file 1 [file Table_1.docx]

Supplementary Table 1. Checklist for Sections in Each Pathology Chapter

|  | **Items Per Pathology Chapter** | **Type of expected material** |
| --- | --- | --- |
|  | 1. **Introduction**   - Introduction to the disease. - Aim of the chapter. | **Literature review** |
|  | **2. Epidemiology**   - Prevalence and incidence data. - Key demographic trends. |  |
|  | **3. Evaluation.**   - Anthropometric Assessments - Biochemical Assessments - Clinical Assessments - Dietetic Assessments - Other Relevant Factors |  |
|  | **4. Intervention**   - Treatment Goals: Clear, measurable objectives. - Dietetic Interventions: Specific dietary plans and strategies. - Nutritional Counseling: One-on-one or group counseling. - Educational Materials: Infografics, videos, or interactive tools. - Other Interventions:   -Motivational strategies.  -Mobile or digital health applications. |  |
|  | **5. Monitoring & Discussion of realistic treatment expectations.** |  |
|  | **6. Monitoring table** | **Formats** |
|  | **7. Medical History Formats**   - Long Format: Detailed patient history. - Short Format: Concise summary. |  |
|  | -Table with prioritized evaluation items |  |
|  | - Table with prioritized diagnoses |  |
|  | **8. Clinical Case Example**  Detailed case study integrating:  -Medical history.  -Diagnosis.  -Intervention and educational materials.  -Monitoring table. |  |
|  | **9. Interdisciplinary Section**  -Key contributions of interdisciplinary teams (medical, psychological, etc.).  -Roles and coordination within the team. |  |
|  | **10. References** |  |
